# Short-term salivary acetaldehyde increase due to direct exposure to alcoholic beverages as an additional cancer risk factor beyond ethanol metabolism

**DOI:** 10.1186/1756-9966-30-3

**Published:** 2011-01-06

**Authors:** Dirk W Lachenmeier, Yulia B Monakhova

**Affiliations:** 1 Chemisches und Veterinäruntersuchungsamt (CVUA) Karlsruhe, Weissenburger Strasse 3, 76187 Karlsruhe, Germany; 2 Department of Chemistry, Saratov State University, Astrakhanskaya Street 83, 410012 Saratov, Russia

## Abstract

**Background:**

An increasing body of evidence now implicates acetaldehyde as a major underlying factor for the carcinogenicity of alcoholic beverages and especially for oesophageal and oral cancer. Acetaldehyde associated with alcohol consumption is regarded as 'carcinogenic to humans' (IARC Group 1), with sufficient evidence available for the oesophagus, head and neck as sites of carcinogenicity. At present, research into the mechanistic aspects of acetaldehyde-related oral cancer has been focused on salivary acetaldehyde that is formed either from ethanol metabolism in the epithelia or from microbial oxidation of ethanol by the oral microflora. This study was conducted to evaluate the role of the acetaldehyde that is found as a component of alcoholic beverages as an additional factor in the aetiology of oral cancer.

**Methods:**

Salivary acetaldehyde levels were determined in the context of sensory analysis of different alcoholic beverages (beer, cider, wine, sherry, vodka, calvados, grape marc spirit, tequila, cherry spirit), without swallowing, to exclude systemic ethanol metabolism.

**Results:**

The rinsing of the mouth for 30 seconds with an alcoholic beverage is able to increase salivary acetaldehyde above levels previously judged to be carcinogenic in vitro, with levels up to 1000 μM in cases of beverages with extreme acetaldehyde content. In general, the highest salivary acetaldehyde concentration was found in all cases in the saliva 30 sec after using the beverages (average 353 μM). The average concentration then decreased at the 2-min (156 μM), 5-min (76 μM) and 10-min (40 μM) sampling points. The salivary acetaldehyde concentration depends primarily on the direct ingestion of acetaldehyde contained in the beverages at the 30-sec sampling, while the influence of the metabolic formation from ethanol becomes the major factor at the 2-min sampling point.

**Conclusions:**

This study offers a plausible mechanism to explain the increased risk for oral cancer associated with high acetaldehyde concentrations in certain beverages.

## Background

Acetaldehyde (ethanal, CH_3_CHO) is a potent volatile flavouring compound found in many beverages and foods [1-3]. In alcoholic beverages, acetaldehyde may be formed by yeast, acetic acid bacteria, and by coupled auto-oxidation of ethanol and phenolic compounds [3]. In a recent study, a large collective of different alcoholic beverages (n > 1500) was evaluated. Beer (9 ± 7 mg/l, range 0-63 mg/l) contained significantly lower amounts of acetaldehyde than wine (34 ± 34 mg/l, range 0-211 mg/l), or spirits (66 ± 101 mg/l, range 0-1159 mg/l) [4].

According to the International Agency for Research on Cancer (IARC), acetaldehyde associated with alcohol consumption is regarded as 'carcinogenic to humans' (IARC Group 1) [5]. Evidence points to the oesophagus, head and neck as principal sites of carcinogenicity of metabolically or microbiologically formed acetaldehyde. A causal link has been found between alcohol consumption and the occurrence of malignant tumours of the oral cavity, pharynx, larynx, oesophagus, as well as of liver, colorectum, and female breast, so that ethanol in alcoholic beverages is also considered to be 'carcinogenic to humans' (IARC Group 1) [6,7].

*In vitro *evidence shows that the acetaldehyde DNA-adduct α-methyl-γ-hydroxy-1,*N*^2^-propano-2'-deoxyguanosine (Cr-PdG) can be formed in response to acetaldehyde concentrations as low as 100 μM [8]. Two separate studies have proven the mutagenic potential of Cr-PdG in either monkey kidney cells [9], or SV40-transformed human fibroblasts [10], where the adducts result in mutant fractions of between 5-11%. In addition, the Cr-PdG adducts can undergo rearrangement in double-stranded DNA, resulting in the formation of DNA-protein cross-links and DNA interstrand cross-links. DNA-protein cross-links are precursor lesions to sister chromatid exchanges, which have been observed to be elevated in human alcoholics [6]. Both DNA-protein cross-links and DNA interstrand cross-links are mechanistically consistent with the generation of chromosomal aberrations, which have also been observed to be elevated in human alcoholics [6]. Acetaldehyde also interferes with DNA repair mechanisms by inhibiting repair enzymes [11].

Apart from the *in vitro *evidence, the link between acetaldehyde and oral cancer is further substantiated by mechanistic evidence in humans deficient in aldehyde dehydrogenase (ALDH) [6,7]. Strong evidence exists to show that the heterozygous genotype (ALDH2*1/*2) contributes substantially to the development of oesophageal cancer related to alcohol consumption, with up to a 12 fold increase in risk seen in heavy drinkers when compared to carriers of the homozygous ALDH2*1/*1 genotype (which encodes the active enzyme) [12,13]. ALDH deficient humans have higher levels of acetaldehyde in their blood but especially in their saliva after drinking alcohol [14-16], and higher levels of acetaldehyde-related DNA adducts have been measured in their lymphocytes [17].

In addition to acetaldehyde metabolism in the gastrointestinal tract and in the liver, the oral and colonic bacterial flora may also contribute considerably to acetaldehyde accumulation [14,15,18-25]; and for humans with active ALDH2 nearly all acetaldehyde found in the saliva was judged to be of microbial origin [15]. For this reason, poor dental status or lack of oral hygiene are associated with a higher risk for cancer of the upper gastrointestinal tract [26-28]. In addition, chronic alcohol abuse leads to atrophy of the parotid glands and reduced saliva flow, which further aids local acetaldehyde accumulation [29].

A quantitative risk assessment using the margin of exposure (MOE) approach has estimated the average exposure to acetaldehyde that is a direct component of alcoholic beverages as being 0.112 mg/kg body weight/day. The MOE was calculated at 498, which is considered a public health concern, and the lifetime cancer risk would be 7.6 in 10 000. Higher risk may exist for people exposed to higher acetaldehyde contamination, as we have found in certain alcoholic beverages, and exposure scenarios indicate risks in the range of 1 in 1000 [30].

Theoretical calculations that assume an equal distribution between the beverage and saliva showed that the residual acetaldehyde concentrations in the saliva after swallowing could be, on average, 195 μM for beer, 734 μM for wine, 1387 μM for spirits, or 2417 μM for fortified wine, which are above levels previously regarded as potentially carcinogenic [4].

The present study was conducted to evaluate acetaldehyde found as a direct component of alcoholic beverages as an additional cancer risk factor to acetaldehyde formed from ethanol. Our aim was to provide experimental data to substantiate the theoretical calculations mentioned above. In addition, we focused on differences between sub-groups of alcoholic beverages, as there are some epidemiological findings pointing to an increased risk of oesophageal cancer due to consumption of specific alcoholic beverages [31].

## Methods

### Experimental design and sampling

The experiments were conducted within the framework of our function as governmental food and alcohol control institution, which includes a chemical-toxicological as well as an organoleptical evaluation of products by a trained panel of assessors. The experiments included only products legally sold on the market of the European Union (EU). Furthermore, the study only included products that had to be organoleptically tested anyway for other reasons, e.g. to check compliance with EU and national regulations (such as regulation (EC) 110/2008 [32]). The CVUA Karlsruhe is permanently permitted by German federal state law to conduct sensory testing of alcoholic beverages in its capacity as governmental control laboratory [33]. Nevertheless, we decided to conduct the study according to the Helsinki Declaration, and informed consent was obtained from every participant (which is normally unnecessary for our taste panels). All assessors met the following criteria: (i) 20 to 60 years old; (ii) no health problems and not taking drugs; (iii) non smokers; (iv) non-denture wearers; (v) no dental problems (annual dentist visits, twice daily toothbrush use). The alcoholic beverages chosen for our experiments were taken from retail trade by governmental food inspectors. The beverages were used as such, no acetaldehyde or any other additives were added to the alcoholic beverages (with the exception of distilled water to dilute some of the beverages). All beverages were checked for compliance with European food law [32]. The alcoholic strength in the beverages was determined according to Ref. [34], acetaldehyde in the beverages was checked according to Refs. [35,36].

The assessors were asked to be abstinent for at least one day prior to the experiment. All experiments were conducted more than 1 hour after the last meal or drink to ensure there is no contamination of saliva with interfering substances. The assessors were also asked to uphold their standard dental hygiene (twice daily toothbrush use), but not to use alcohol-containing mouthwashes, and not to ingest alcohol-containing foodstuffs during the trial period. Compliance to these criteria including the study selection criteria was obtained in writing by all participants.

The alcoholic beverages were rinsed by the assessors in their mouths for 30 sec and then spit out similar to a wine tasting (no ingestion or swallowing was allowed). Saliva was sampled prior to rinsing, as well as 30 sec, 2 min, 5 min and 10 min after spitting-out. Sampling was conducted using the saliva collection system salivette^® ^(Sarstedt, Nümbrecht, Germany). The system consists of cotton swabs that are gently chewed by the assessors. Afterwards, the swab is replaced in the suspended insert of the salivette^®^, which is firmly closed using a stopper. The saliva is recovered by centrifugation of the salivette^® ^at 1,000 g for 2 min. The clear saliva supernatant was used for acetaldehyde analysis.

### Analytical procedure

The determination of acetaldehyde in saliva samples was conducted using either enzymatic analysis or gas chromatography. The enzymatic analysis was conducted with aldehyde dehydrogenase according to the method of Lundquist [37,38], which is available as commercial test-kit (acetaldehyde UV-method, Cat. No. 0668613, R-Biopharm, Darmstadt, Germany). The detection limit of the assay is 0.25 mg/l (5.6 μmol/l). For further details about the method see Beutler [39].

The test-kit instructions of the manufacturer were followed without modification. 0.2 ml of saliva supernatant were used as sample solution. The enzymatic measurement was conducted immediately (within 1 hour) after saliva sampling to exclude losses of acetaldehyde due to evaporation or oxidation. The spectrophotometric measurements were performed on a Perkin Elmer Lambda 12 dual beam spectrometer equipped with automatic cell changer, which allows the software-controlled measurement of a sample series (n = 13) without manual intervention.

The procedure for the gas chromatographic (GC) analysis was previously described in detail for the determination of acetaldehyde in saliva after alcohol-containing mouthwash use [40]. Both the enzymatic and the GC procedure were validated for the use to determine saliva after alcoholic beverage use, which leads to higher concentrations than used in our previous validation after mouthwash use [40]. Artefactual acetaldehyde formation was excluded by analyzing blank samples (i.e. saliva before alcohol use) with addition of 50 μl of pure ethanol. All samples were below the detection limit of both the enzymatic and GC method, no artefactual acetaldehyde was formed. The method was further validated using authentic saliva samples after alcohol use (2 min). Saliva samples of five samplings were pooled and homogenized as quality control sample. The quality control sample (250 μM) was then analyzed for five times with each method. The precision of the method expressed as coefficient of variation (CV) was 9.7% (GC) and 10.3% (enzymatic method). The recovery of the method was determined by spiking blank saliva samples with acetaldehyde (n = 6). The recovery was 102.2 ± 2.9% for GC and 103.3 ± 5.9% (enzymatic method). As most of the samples were above 50 μM, we have not investigated the detection limits and only investigated a range above 20 μM, which was the lowest calibrator. The results of both methods were not significantly different and both methods were judged suitable for the purpose of analyzing saliva samples for acetaldehyde. While the GC method is more precise, sensitive and selective, we used the enzymatic assay for approximately half of the samples to be analyzed, because of its lower costs and faster analysis times.

### Statistics

All data were evaluated using Unscrambler X version 10.0.1 (Camo Software AS, Oslo, Norway) and Origin V.7.5 (Originlab, Northampton, USA). Data are summarized as means and standard deviations between assessors for each data point. Statistical dependence between alcoholic strengths and the acetaldehyde contents of the beverages and the salivary acetaldehyde were evaluated using multiple linear regression (MLR) and Analysis of Variance (ANOVA) for all time data points (30 sec, 2 min, 5 min, and 10 min). The regression analysis was also conducted with the area under the curve (AUC) for the complete time period under investigation (0-10 min). Statistical significance was assumed at below the 0.05 probability level.

## Results

Table 1 shows the alcoholic strengths and acetaldehyde contents of the alcoholic beverages, as well as the resulting average salivary acetaldehyde concentrations for the assessors. The assessors (up to n = 10 per beverage, see Table 1) had an average age of 27 ± 6 years and 70% were female. The highest salivary acetaldehyde concentration was found in the saliva 30 sec after using the beverages in all cases, and the average content was 353 ± 164 μM (range: 56-1074 μM). The acetaldehyde level then decreased at the 2-min sampling (156 ± 46 μM, range: 41-337 μM), the 5-min sampling (76 ± 19 μM, range 26-131 μM) and at the 10-min sampling (40 ± 18 μM, range: n.d.-94 μM). The inter-individual variation in salivary acetaldehyde content is relatively high, with an average CV of 48% between assessors. No apparent gender or age related differences were seen, however, due to the relatively homogenous ages of the probands, the statistical power does not allow to make a definite conclusion on an effect of age. Similarly, no statistically significant conclusion on the effect of gender can be gathered from the data.

**Table 1:** Alcoholic strength and acetaldehyde content of alcoholic beverages and the resulting salivary acetaldehyde concentrations

				Salivary acetaldehyde [μM]^a^			
				
Alcoholic beverage	Alcoholic strength [% vol]	Acetaldehyde^b^ [μM]	Number of assessors^f^	0.5 min	2 min	5 min	10 min
Beer^c^	5	210	1	98 ± 4	113 ± 13	44 ± 6	n.d.^e^

Cider^c^	5.5	2529	4	428 ± 159	202 ± 72	70 ± 41	26 ± 7

Wine^c^	13	474	3	315 ± 288	225 ± 117	115 ± 62	39 ± 30

Calvados^d^	15^g^	411	2	93 ± 59	51 ± 16	27 ± 10	n.d.^e^

Sherry^c^	15	2583	3	291 ± 117	114 ± 77	68 ± 25	n.d.^e^

Vodka^d^	16^g^	n.d.	3	56 ± 11	59 ± 30	36 ± 27	n.d.^e^

Calvados^c^	40	1095	2	194 ± 70	134 ± 5	91 ± 7	68 ± 37

Vodka^d^	40	n.d.	2	220 ± 185	125 ± 87	96 ± 81	83 ± 64

Vodka^c^	40	n.d.	10	116 ± 31	86 ± 61	67 ± 25	21 ± 21

Grape marc spirit^d^	40	11120	1	231 ± 137	41 ± 32	26 ± 12	32 ± 15

Grape marc spirit^d^	40	9444	2	554 ± 359	187 ± 116	46 ± 10	94 ± 100

Tequila^c^	40	530	1	143 ± 54	164 ± 35	131 ± 47	59 ± 18

Grape marc spirit^c^	41	15197	4	1074 ± 399	256 ± 117	90 ± 60	58 ± 39

Grape marc spirit^d^	41	15851	3	625 ± 231	243 ± 211	103 ± 71	86 ± 69

Cherry spirit^c^	43	8522	1	856 ± 17	337 ± 42	123 ± 25	41 ± 9

^a ^Salivary acetaldehyde before use was not detectable (< 20 μM) in all cases. Average and standard deviation of all assessors are shown (in the case of n = 1, the average and standard deviation of the two replications per assessor are shown).

^b ^Acetaldehyde directly contained in the alcoholic beverage as determined with GC analysis.

^c ^Enzymatic analysis of salivary acetaldehyde.

^d ^GC analysis of salivary acetaldehyde.

^e ^Not detectable (< 20 μM).

^f ^Two replications were conducted with each assessor on different days.

^g ^Dilution of a commercial product at 40% vol with distilled water

**Figure 1:**
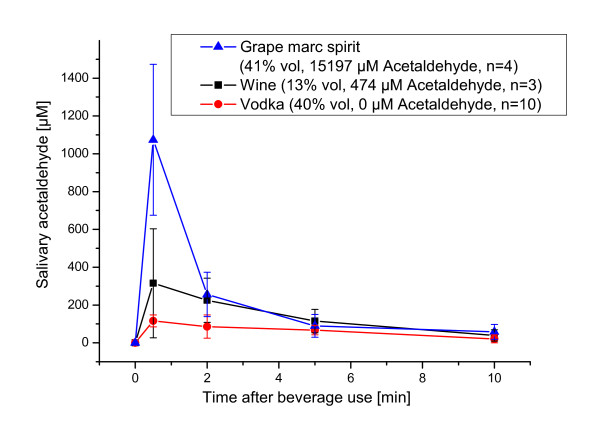
**Salivary acetaldehyde concentrations after alcoholic beverage use in three different samples**. The values are average and standard deviation of all assessors. The figure legend states the alcoholic strength (in % vol) and the acetaldehyde content (in μM) in the beverages, as well as the number of assessors used for each beverage.

Figure 1 shows typical profiles for three beverages with different alcoholic strengths and acetaldehyde contents. The attempt to build univariate linear models between either the values of alcoholic strengths or acetaldehyde in the beverages and salivary acetaldehyde concentrations was unsuccessful. This finding was consistent for any of the calculation methods (for AUC or for the specific time points). Thus, the acetaldehyde concentration in saliva clearly did not depend on only one parameter. We therefore used multilinear regression (MLR) to evaluate the combined influence of ethanol and acetaldehyde in the beverages.

**Figure 2:**
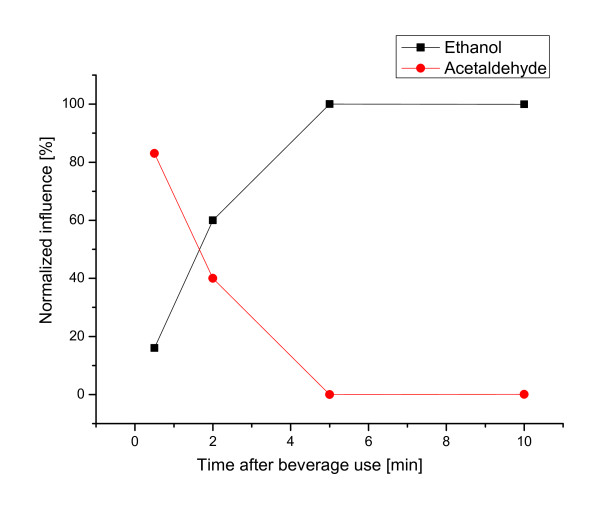
**Influence of ethanol and acetaldehyde content of the beverages on the salivary acetaldehyde concentration**.

The results of ANOVA for the MLR calculations are summarized in Table 2. ANOVA suggests that both global models (for the independent time points and AUC) are significant. Table 2 also provides ANOVA results for the significance of individual effects on salivary acetaldehyde concentrations for each time point. At the first time-point (30 sec), acetaldehyde that directly comes from the beverages dominates in the saliva. Only a minor influence of the ethanol content was evident during the first 30-sec after beverage use, but it then gradually increased with an almost 100% influence from the 5 min time point (Figure 2).

**Table 2:** ANOVA results for multiple linear regression (MLR) models

	Model for individual time points^a^				Model for AUC
		
	0.5 min	2 min	5 min	10 min	
R	0.80				0.81

p (Model)	0.0022				0.0030

p (Ethanol)	0.9400	0.9200	0.1200	0.0098	0.3400

p (Acetaldehyde)	0.0002	0.0190	0.9900	0.3500	0.0057

^a ^time after beverage use

## Discussion

Our results confirm the observation of high inter-individual variations in the acetaldehyde levels in saliva following ethanol exposure previously noted during *in vitro *and *in vivo *experiments. These high variations were judged to be predominantly caused by the differences in acetaldehyde production capacity among the oral bacteria [19,40,41]. While our assessor collective was too small for statistical investigation of sub-collectives, we can nevertheless qualitatively confirm the *in vitro *results of Ernstgård [41], as we saw no apparent gender or age related differences. The small sample size of assessors (for some of the beverages only n = 1) is also a major limitation of the study. A further limitation of the study includes the use of the salivette^® ^saliva collection method, which may stimulate salivary secretion and thus dilute acetaldehyde and ethanol concentrations. Our study therefore could underestimate rather than overestimate the risk.

In our previous experiments on acetaldehyde in saliva after use of alcohol-containing mouthwashes [40], we did not detect any dependence between salivary acetaldehyde and ethanol or acetaldehyde concentration of the mouthwashes. However, the concentrations of both compounds were lower in the mouthwashes than in the alcoholic beverages under investigation in the present study and the previous study design had only low statistical power. This explains that this time within our resources to analyze around 500 samples, our aim was to rather sample a larger number of beverages with fewer assessors than vice versa, leading to increased variance of ethanol and acetaldehyde contents in the beverage collective and similarly increased power for the statistical calculations on these parameters. Nevertheless, we were still surprised that a statistically significant dependence occurs in this case of alcoholic beverages. In the mouthwashes (which contained very little acetaldehyde), the metabolically produced acetaldehyde was the predominant factor for salivary acetaldehyde [40]. In contrast, in the case of alcoholic beverages, salivary acetaldehyde is characterized by both the acetaldehyde contained in the beverage and that formed from ethanol.

The influence of the directly contained acetaldehyde, however, is short-term and only prevails during the first 2 minutes after rinsing of the mouth with an alcoholic beverage for 30 seconds. Subsequently, the concentration depends on the amount of ethanol available for metabolic oxidation. Further research should be conducted to clarify the influences in the time period between 30 sec and 5 min in more detail, as our approach does not allow to interpolate the exact time at which the change between the two factors occurs.

Similar findings to our study were generally made by Yokoyama et al. [16], with a slightly different experimental design that used ingestion of different alcoholic beverages up to the same blood alcohol concentration. In this study, similar to our findings, the type of alcoholic beverages had no effect on the saliva acetaldehyde concentration 30 minutes or more after drinking, while a beverage dependency was observed directly after the completion of drinking (the period between 0 and 30 min was not further investigated by the authors, however). Apart from the ingestion used, our results are not directly comparable to those of Yokoyama et al. [16] as they used spirits that had all been diluted to 13% vol. Our collective of alcoholic beverages also generally contained higher levels of acetaldehyde, as we intentionally selected beverages with high contamination status for the experiment, in order to increase the likelihood of observing a significant effect when compared to non-contaminated vodka. The limitation of the comparably low sample size in our study must also be kept in mind. Our results are therefore not generalizable for a population-based risk assessment, as the beverages are not representative of those available in the market. The contamination status of the beverages also explains the extremely high salivary acetaldehyde concentrations up to over 1000 μM, which were never before described in the literature, not even for ALDH2-deficient subjects [14,16,19,42,43]. Our *in vivo *results confirm our previous theoretical calculations of potentially high short-term acetaldehyde concentrations, as mentioned in the introduction, which were deduced from typical levels found in beverages [4].

This now leaves the question regarding how to interpret the health effects of this short-term high exposure to acetaldehyde. Whether a threshold for the carcinogenicity of acetaldehyde exists is still debatable and its potential magnitude is unclear [40]. The natural acetaldehyde background levels in human blood are very low and generally not detectable (< 0.5 μM) [44] and the endogenous salivary acetaldehyde levels are assumed to be likewise, as they are below 1 μM [40]. This assumption was recently confirmed *in vitro*, as an average of 0.3 μM acetaldehyde occurred in 36 saliva samples without ethanol exposure [41]. The lowest concentration of acetaldehyde that has induced sister chromatid exchange in Chinese hamster ovary cells *in vitro *(3.9 mg/l, 88 μM) in a study of Obe and Ristow was suggested as threshold for toxicity evaluation [45]. This is in agreement not only with the 100 μM threshold for Cr-PdG formation [8], but also with indirect evidence on salivary acetaldehyde concentration provided by human studies on alcohol consumption. After a moderate dose of alcohol, acetaldehyde levels in the saliva range between 18 and 143 μM within 40 minutes of alcohol ingestion [19]. After ingestion of a moderate dose of alcohol, ALDH2-deficient Asians have detectable acetaldehyde levels in their saliva that are 2-3 times higher than in Asians with the normal enzyme. This is associated with a remarkably increased risk for digestive tract cancers [14]. Salaspuro recently summarized all of this evidence and estimated that the mutagenic amount of acetaldehyde in saliva falls between 50 and 150 μM [46]. Linderborg et al. [31] indicated that the oral and upper digestive tract mucosa is exposed to a much higher acetaldehyde concentration after ingestion of calvados (i.e., 20-50 times higher than those considered to be mutagenic), which is consistent with our results.

## Conclusions

Because alcohol use significantly increases salivary acetaldehyde above endogenous levels (even if the alcohol is not contaminated, as in the case of vodka), we ascertain that a "biological threshold" is clearly exceeded during alcohol consumption. The observations of the present study and the suggested molecular mechanisms could conceivably explain the increased oral cancer risk associated with alcohol use seen in epidemiological studies [6]. Salivary acetaldehyde concentrations in the range associated with sister chromatid exchange and Cr-PdG formation are clearly achievable. Highly contaminated beverages could present a higher cancer risk than beverages with none or very low concentrations of acetaldehyde (for example, see Linderborg et al. [31]). Currently only limited and inconclusive epidemiological evidence exists to confirm this beverage specificity, however. From the 56 studies on oesophageal cancer summarized by IARC [6], the influence of the type of alcoholic beverage consumed was examined in several studies. Consumption of beer or hard liquor led to a higher relative risk than consumption of wine [47-52], whereas two studies [53,54] also found an excess risk for wine drinkers. Most of the studies that investigated types of alcoholic beverage showed no substantial difference in risk [6]. This probably derives from the fact that the most commonly consumed beverage groups on a population scale (i.e., beer, wine and white spirits) are typically low in acetaldehyde content. It would be also challenging to design an epidemiological study that could consider the acetaldehyde content, when even the ethanol amount is often difficult to measure in retrospect [55] and international data on acetaldehyde content of alcoholic beverages are very limited [4].

Currently, the acetaldehyde content of most alcoholic beverage types is not regulated. The recent IARC evaluation of acetaldehyde associated with alcohol consumption as a "group 1" carcinogen has not yet been implemented in international risk assessments (e.g., by JECFA or EFSA). Until such assessments become available, we would currently recommend the implementation of the ALARA principle ("as low as reasonably achievable") [56]. In the case of spirits, which were linked to very high short-term acetaldehyde concentrations in our study, avoidance of acetaldehyde contamination is relatively easy if the first distillation fractions are discarded [4].

## Competing interests

The authors declare that they have no competing interests.

## Authors' contributions

DWL conceived of the study, coordinated the work, and drafted the manuscript. YBM conducted the statistical calculations, and composed the tables and figures. All authors read and approved the final manuscript.
